# Heavy Metals and Essential Elements in Association with Oxidative Stress in Women with Polycystic Ovary Syndrome—A Systematic Review

**DOI:** 10.3390/antiox12071398

**Published:** 2023-07-07

**Authors:** Tinkara Srnovršnik, Irma Virant-Klun, Bojana Pinter

**Affiliations:** 1 Division for Women’s Healthcare—Šiška Unit, Community Health Centre Ljubljana, Metelkova Ulica 9, 1000 Ljubljana, Slovenia; tinkara.srnovrsnik@zd-lj.si; 2 Faculty of Medicine, University of Ljubljana, Vrazov Trg 2, 1000 Ljubljana, Slovenia; 3 Clinical Research Centre, University Medical Centre Ljubljana, Vrazov Trg 1, 1000 Ljubljana, Slovenia; irma.virant@kclj.si; 4 Division of Obstetrics and Gynecology, University Medical Centre Ljubljana, Šlajmerjeva 3, 1000 Ljubljana, Slovenia

**Keywords:** PCOS, trace elements, toxic metals, chronic inflammation, oxidative damage, endocrine disruptors

## Abstract

Altered levels of heavy metals and essential elements have been associated with oxidative stress (OS) and metabolic and hormonal changes in women with polycystic ovary syndrome (PCOS). We aimed to summarize the knowledge on the association of heavy metals and essential elements with OS in PCOS. An electronic literature search using PubMed for studies published between January 2008 and April 2023 was conducted. We evaluated heavy metals and essential elements in relation to OS in PCOS in 15 articles. PCOS women had increased antimonium (Sb), cadmium (Cd), lead (Pb), mercury (Hg), arsenic (As), tellurium (Te), thallium (Tl) and osmium (Os) blood levels and decreased zinc (Zn) blood levels; the results of copper (Cu) blood levels were conflicting. Some studies showed a significant correlation between heavy metals (Sb, Cd, Pb, Hg, As, Te and Tl) and essential elements (Se, Zn, Cr, Ca, Mg and Cu) and markers of OS and chronic inflammation. Heavy metals (Sb, Cd, Pb and Hg) and essential elements (Zn, Cr, Se, Ca, Mg and Cu) were associated with metabolic and hormonal characteristics in PCOS. There might be a possible benefit from supplementation therapy in reducing OS and endocrinological problems related to PCOS. Our review confirmed an association between heavy metals and essential elements with OS in PCOS women. This systematic review is registered in PROSPERO under number CRD42023418453.

## 1. Introduction

Polycystic ovary syndrome (PCOS) is one of the most common endocrinopathies with a multifactorial aetiology, affecting 5–10% women of reproductive age [1]. Regarding the consensus meeting organized by the European Society of Human Reproduction and Embryology (ESHRE) and the American Society for Reproductive Medicine (ASRM), a diagnosis of PCOS is given if two out of the following three criteria are fulfilled: (i) clinical and/or biochemical hyperandrogenism, (ii) chronic oligomenorrhea and/or anovulation or (iii) the presence of polycystic ovaries in transvaginal ultrasonography [2].

### 1.1. PCOS and Chronic Inflammation

Metabolic disorders in PCOS, such as insulin resistance (IR), diabetes and obesity, can be explained by the existence of a vicious circle of effects. Chronic hyperandrogenaemia results in abdominal adiposity which favours hypoadiponectinemia, adipose tissue dysfunction and cytokine excess. Abdominal adiposity further facilitates androgen excess directly with the ovarian or adrenal response to inflammatory mediators or indirectly by the development of IR, since insulin facilitates androgen secretion by these glands [3]. In PCOS women, increased levels of C-reactive protein (CRP), interleukin 18 (IL-18), tumour necrosis factor (TNF-α), interleukin-6 (IL-6) and ferritin or white blood cell count, and decreased levels of anti-inflammatory cytokines, such as adiponectin and omectin, were found [4]. Therefore, PCOS is considered a pro-inflammatory state with low-grade chronic inflammation interlinking obesity, insulin resistance, diabetes and cardiovascular disease due to endothelial disfunction [5,6].

### 1.2. PCOS and Oxidative Stress

In 2006, González et al. indicated that ROS generation in response to hyperglycemia is increased in PCOS, independent of obesity contributing to a proinflammatory state that induces IR and hyperandrogenism [7]. This was confirmed in a meta-analysis by Murri et al., where circulating markers of OS were altered in PCOS women independent of weight excess [3]. OS refers to the imbalance between the oxidation and antioxidation system resulting in an altered redox state of cells. The accumulation of active oxidation substances can damage DNA, proteins, lipids, carbohydrates and other molecules [8,9]. The two main types of oxidative active molecules are (1) reactive oxygen species (ROS) and (2) reactive nitrogen species [8]. The two main types of antioxidants include (1) enzymatic antioxidants (e.g., superoxide dismutase (SOD), glutathione peroxidase (GPx), catalase (CAT) and glutathione reductase) and (2) non-enzymatic antioxidants (e.g., vitamin C, vitamin E, β-carotene, selenium, zinc, glutathione (GSH) and ferritin) [10,11]. OS and chronic inflammation are closely inter-related as inflammation induces generation of ROS, while OS promotes and aggravates inflammation [6]. The cross-links between OS, IR, hyperandrogenemia and obesity remain complicated. IR encourages OS because hyperglycemia and higher levels of free fatty acid lead to reactive oxygen species (ROS) production when entering the cells. This results in a large number of reducing metabolites which are transferred into mitochondria for oxidization which finally lead to increased ROS production [12]. On the other hand, OS impairs glucose uptake in muscle and adipose tissue and reduces insulin secretion from pancreatic β cells [3]. Adipose macrophages in overweight and obese PCOS women promote OS and low-grade inflammation and block the signal transduction of insulin [8]. Since OS has also been reported in non-obese PCOS women, obesity is not the only factor leading to increased OS in PCOS [12,13,14]. Several antioxidant drugs have been studied in PCOS women, e.g., resveratrol, green tea, curcumin, coenzyme Q10 and astaxanthin [15].

**Figure 1:**
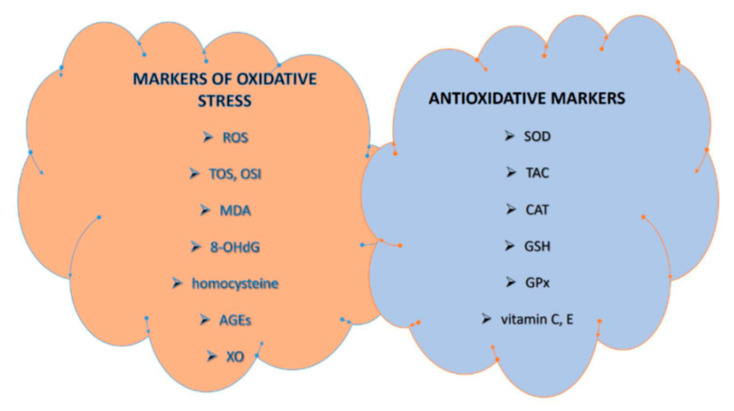
Commonly used markers of OS in PCOS. Abbreviations: AGEs, advanced glycation end products; CAT, catalase; 8-OHdG, 8-hydroxydeoxyguanosine; GPx, glutathione peroxidase; GSH, glutathione; MDA, malondialdehyde; OSI, oxidative stress index (ratio of TOS to TAC); ROS, reactive oxygen species; SOD, superoxide dismutase; TAC, total antioxidant capacity; TOS, total oxidant status; XO, xanthine oxidase.

There is discrepancy and no consensus on the set of standardized markers and measurement units of OS in clinical studies to facilitate comparison between them [16,17]. Commonly used markers for assessment of OS in PCOS are summarized in Figure 1 [11,16,17,18].

### 1.3. PCOS, Metal Exposure and Oxidative Stress

The serum levels of heavy metals and essential elements can change in PCOS women [19]. The mechanism underlying heavy metal toxicity in humans is mainly their interaction with the sulfhydryl groups in the non-enzymic antioxidant system (e.g., replacing a hydrogen atom on the reduced GSH moieties), resulting in the formation of organo-metallic complexes which deactivate further biochemical reactions [20]. Hormonal and metabolic effects (e.g., diabetes and increased body mass index (BMI)) are associated with heavy-metal-induced OS and subsequent decreased insulin gene promoter activity in pancreatic β cells and changes in human gonadotropin and reproductive hormone levels [21,22,23]. Although essential elements are crucial for normal cellular function and incorporation into many metalloenzymes and proteins responsible for the regulatory pathways of OS, evidence suggests the association of altered levels of essential elements with metabolic syndrome and PCOS [24]. Hence, the effects of some essential elements are associated with insulin action, glucose metabolism, cytokine production, inflammation, immune defense and oxidative stress [25].

Below we describe some of the metals associated with OS in women with PCOS.

#### 1.3.1. Heavy Metals

Lead (Pb) is used in industry in the production of batteries, cables, pigments, chemical additives and petrol. The general population is exposed via the ingestion of contaminated food and water and inhalation of airborne Pb [26]. In rodents, Pb has been associated with altered steroidogenesis, follicular growth and maturation and decreased gonadotropin binding and serum gonadotropin levels, while in humans, antiestrogenic effects were observed [27]. Lead might play a role in the pathogenesis of PCOS by depleting GSH and protein-bound sulfhydryl groups and enhancing lipid peroxidation [19].

Cadmium (Cd) is widely used in the production of pigments, batteries and fertilizers. Exposure in the general population usually occurs by ingestion of contaminated food and water, contact with consumer products containing Cd (e.g., nickel/cadmium batteries, pigments, paints and plastic products) or with tobacco smoking [26,28]. In rodents, Cd has been associated with a disrupted hypothalamic-pituitary-gonadal axis, altered steroidogenesis and decreased gonadotropin binding and serum gonadotropin levels [27,28]. In humans, Cd expresses estrogenic effects [27] and is associated with increased serum follicle-stimulating hormone (FSH) [23] and a disruption of the pancreatic islet β-cell function [22].

The predominant sources of arsenic (As) are agricultural products, the foundry industry and combustion of fossil fuels. Exposure in the general population occurs mainly through ingestion of contaminated food and water [26]. In mammals, As might impair the glucose metabolism and insulin secretion in β-cells [22]. In humans, As-induced OS can lead to apoptosis of pancreatic β-cells and a decrease in insulin secretion [22,29].

Mercury (Hg) is widely used in foundry, mining, manufacturing industries and in electrical instruments and medical products (e.g., thermometers, thermostats, dental amalgams, switches and batteries). Exposure in the general population occurs mainly through ingestion of contaminated food and water, especially with fish consumption [26]. In humans, Hg impairs the antioxidant system, leading to increased lipid peroxidation and OS, while in mice, OS might be involved in β-cell apoptosis, resulting in hyperglycaemia [30].

Antimony (Sb) and its compounds are naturally present in the earth’s crust; in industry, Sb is used in the production of semiconductors, infrared detectors and diodes, but some Sb compounds have been used in the treatment of leishmaniasis and schistosomiasis. Exposure to Sb in the general population occurs mostly by ingested food and water [31]. In vitro studies on human cells suggested possible DNA damage by inhibition of the enzymes involved in DNA repair and OS from lipid peroxidation and thiol compound interactions; increased oxidative DNA damage was observed in workers exposed to antimony trioxide [32].

Tellurium (Te) is a rare, toxic element that is found in the earth’s crust. It is used in copper alloys, stainless steel and to colour glass and ceramics and has been associated with the development of new materials such as CdTe probes, photovoltaic products and other compounds used in nanotechnology. Routes of exposure can be ingestion by food or by inhalation of its aerosol [33]. The toxicity of Te and its inorganic derivatives is assumed to be the consequence of the strong oxidizing properties resulting in deleterious ROS, but on the other hand, organic tellurides have exerted antioxidant properties in in vitro studies [34].

Thallium (Tl) is a toxic metal, with toxicity much higher than Hg, Cd and Pb. The source of contamination is mining, natural weathering, ore combustion or smelting, electronic materials, alloy manufacturing and medical diagnosis. Human exposure is usually due to ingestion of contaminated food and water, inhalation of contaminated dust and fumes or skin absorption [35]. In vitro studies indicate that Tl increases ROS contents by impairing the mitochondrial function, while in animal models of Tl intoxication, high amounts of lipid oxidation end-products were found in the brain [36].

Osmium (Os) is a precious white heavy metal unlikely to cause toxicity. However, osmium tetroxide, its oxidation product, is a strong oxidizing agent that vaporizes easily and sublimates at room temperature. It may cause severe burns to the eyes, skin and respiratory and gastrointestinal tract. Osmium tetroxide is used as a fat fixing and staining agent for adipose tissue in laboratories, in photography and as a catalyst in organic synthesis [37]. The reproductive toxicity of osmium tetroxide in humans remains unexplained.

#### 1.3.2. Essential Elements

Selenium (Se) is a microelement found in the foodstuff of both plant and animal origin. As a constituent of selenoproteins, it exhibits anti-inflammatory characteristics. It acts antagonistically to heavy metals such as As, Cd, Pb and Hg [38]. In humans, Se is a constituent of active centres of antioxidant enzymes GPx, thioredoxin reductase and iodothyronine deiodinase. It reduces adverse processes of lipid peroxidation and protects against DNA damage [39]. In vitro and in vivo studies have shown that Se possess insulin-like actions, suggesting that Se could affect carbohydrate and fat metabolism [40].

Magnesium (Mg) is an essential macromineral and one of the most important intracellular cations abundantly found in human body, particularly in bones (about 60%). It is involved in numerous biochemical reactions that regulate carbohydrate, fat and protein metabolism [41]. It improves insulin receptor sensitivity and facilitates cellular glucose transportation. In humans, it modulates the release of proinflammatory cytokines and mediates the release of anti-inflammatory cytokines and OS. Secondary to hypomagnesemia, it has been associated with the reduced activity of antioxidant enzymes and the activation of inflammatory pathways [42].

Zinc (Zn) is an essential trace mineral, predominantly presented intracellularly, and is an important element for hormonal and islet functions, glucose homeostasis and promotion of the stability and binding ability of insulin receptors [24]. Zn is a cofactor of antioxidant enzymes such as CAT and SOD and plays an essential role in mitochondrial oxidative stress. It counteracts oxidation by occupying binding sites for iron and copper in lipids, proteins and DNA, thus reducing oxidative damage in various human cells [43,44,45].

Copper (Cu) is an essential trace element and a cofactor of many enzymes involved in redox reactions, such as cytochrome c oxidase or in association with Zn as a Cu-Zn SOD [46,47]. In addition to its enzymatic roles, Cu can induce OS by catalysing the formation of ROS and decreasing GSH levels [46]. Therefore, chronic copper overload can result in oxidative damage [47].

Trivalent chromium (Cr) has been considered as an essential trace element, but recent studies indicate that this status should be removed as the effects of Cr appear to be pharmacological rather than nutritional [48]. Cr has been associated with insulin action and glucose metabolism [25]. In a high glucose-treated human erythrocyte model, Cr reduced protein glycosylation and lipid peroxidation. It has been speculated that Cr may reduce OS by activation of GPx or some other antioxidative enzymes [49]. Although Cr supplementation may improve OS parameters according to a meta-analysis by Morvaridzadeh [50], some novel studies warrant caution in recommending Cr supplementation due to its association with OS, DNA damage, genomic instability and carcinogenicity [41,51].

Calcium (Ca) is an important material of many indispensable molecules such as 25-hydroxyvitamin D and is essential to body organisation and structure [47]. Elevation of Ca is required for insulin secretion and Mg deficiency can secondarily lead to changes in cellular Ca levels [46]. Ca and vitamin D have been suggested to act jointly rather than alone. Ca intake may affect OS through Ca transport and signalling lines [52], while vitamin D supplements might have beneficial effects on OS by improving cellular GSH levels and decreasing ROS production and levels of pro-inflammatory factors, as demonstrated in in vitro studies [53]. Intensive Ca entry and OS induce neutrophil activation [54].

Studies that have investigated the potential association of heavy metals and essential elements with OS in women with PCOS are scarce. Therefore, the aim of our systematic review was to review the current knowledge on possible associations of metals with OS in PCOS. This systematic review is registered in PROSPERO under number CRD42023418453.

## 2. Materials and Methods

### 2.1. Literature Identification

This systematic review is reported in accordance with the Preferred Reporting Items for Systematic Reviews and Meta-analyses (PRISMA) guideline. The PRISMA checklist is provided in the Supplementary Materials. We conducted an electronic literature search using the National Library of Medicine (PubMed) database. The following medical subject heading (MeSH) terms, keywords and their combinations were used to search for the studies reporting on the role of heavy metals and essential elements in the occurrence of oxidative stress in PCOS in women: »polycystic ovary syndrome «OR» PCOS «AND» oxidative stress «AND» metals«. The database was searched for studies published from January 2008 to 1 April 2023.

### 2.2. The Inclusion and Exclusion Criteria

The inclusion criteria consisted of randomized control studies, case–control studies and prospective and descriptive studies. We excluded studies not on humans, reviews and studies published in languages other than English. To detect other relevant trials, we performed a hand search of the reference lists of full-text articles that met our criteria in the primary literature search. Each author assessed each article independently. To determine the final eligibility, the authors separately reviewed titles, abstracts and full-text articles. The selected articles were read in full to confirm eligibility and to extract data. Disagreements were resolved through scientific discussion, if necessary.

### 2.3. Data Extraction

From the included studies, the following information was extracted for detailed evaluation: characteristics of the included studies (first author, year of publication and country), study design characteristics (study design, sample type and methods including statistics), sample size, type of metal studied and main findings.

## 3. Results

### 3.1. Literature Search

**Figure 2:**
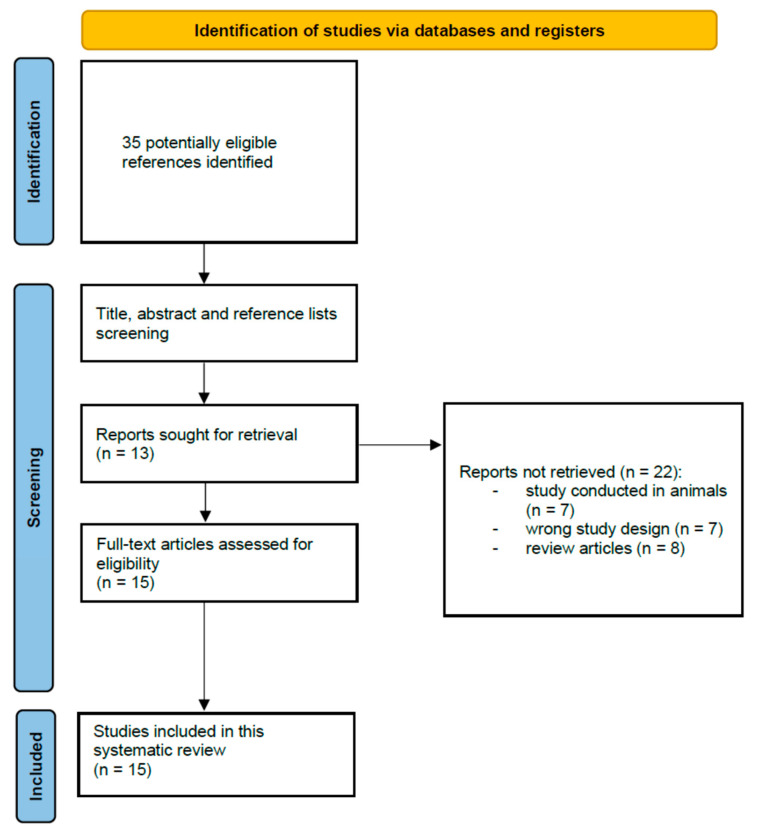
PRISMA flow diagram of the study selection process. Association of heavy metals and essential elements with oxidative stress in polycystic ovary syndrome.

After the primary literature search, we identified 35 potentially eligible citations. Based on our inclusion criteria, we selected 13 studies for initial screening. We screened the reference lists of primarily selected studies and removed duplicates, review articles and studies not conducted in humans and in languages other than in English. Finally, 15 suitable studies met our inclusion criteria (Table 1). Figure 2 presents a PRISMA flow diagram of the association of heavy metals and essential elements with oxidative stress in PCOS.

### 3.2. Association of Heavy Metals and Essential Elements with Oxidative Stress in Women with PCOS

In Table 1, the results of the systematic review on the association of heavy metals and essential elements with oxidative stress in PCOS are summarized. Statistically significant differences are presented.

#### 3.2.1. Heavy Metals and Oxidative Stress in Women with PCOS

In this literature review, three studies studied an association of heavy metals with OS in PCOS women when compared to controls [66,68,69]. Increased serum levels of As in a PCOS population (mean level 2.68 ± 0.50 ppb vs. 1.95 ± 0.34 ppb, respectively) were found in a study by Abudawood et al. [68], but not in a study by Kirmizi et al. (6.5 (2.5–10) vs. 6.4 (3.7–14.1) ppb, respectively) [66]. Higher serum levels of Sb in PCOS women were found in two studies (3.1 (2.4–13.3) vs. 2.9 (1.8–4.7) ppb and 2.5 ± 0.23 vs. 1.89 ± 0.31 ppb, respectively) [66,69]. Women with PCOS also had elevated serum levels of Cd (1.2 (0.7–6) vs. 0.7 (0.4–4.5) ppb and 1.75 ± 0.44 vs. 0.59 ± 0.22 ppb, respectively) [66,68], Pb (23.1 (11.6–90.1) vs. 15.5 (9.3–56.7) ppb and 83.19 ± 14.4 vs. 36.69 ± 6.57 ppb, respectively) [66,68], Hg (2.2 (0.6–3.8) vs. 1.3 (0.4–2.5) ppb and 14.55 ± 2.99 vs. 5.0 ± 1.08 ppb, respectively) [66,68] and Tl, Te and Os (12.69 ± 1.05 vs. 1.41 ± 0.4 ppb, 12.33 ± 1.31 vs. 1.32 ± 0.46 ppb and 13.0 ± 0.97 ppb vs. 1.51 ± 0.45 ppb, respectively) [69].

Regarding markers of OS and chronic inflammation in PCOS population, Kirmizi et al. found lower serum total antioxidant status (TAS), oxidative stress index (OSI) and SOD values and a higher total oxidant status (TOS), MDA, high sensitivity CRP (hs-CRP) and tumour necrosis factor alpha (TNF-α). There was a positive correlation of Sb and Pb with MDA and markers of chronic inflammation, and a negative correlation with TAS, OSI and SOD. Cd appeared to be negatively correlated only with TAS [66]. In a study by Abudawood et al., there was a negative correlation of As, Pb and Hg with GSH levels in a PCOS population. Additionally, As and Pb were negatively correlated with SOD levels [68]. In another study by Abudawood et al., the PCOS group had decreased serum levels of TAC that appeared to be negatively correlated with Te, Tl, Sb and Os [69].

Metabolic parameters in PCOS women were evaluated in two studies [66,68]. Sb was positively correlated with HOMA-IR and fasting blood glucose (FBG) [66]. There was a positive correlation of Cd with BMI, HOMA-IR, FBG, insulin levels [66] and total cholesterol [68]. Pb positively correlated with FBG [66], while Hg positively correlated with FBG and glycated haemoglobin (HbA1c) [68]. When evaluating the waist/hip ratio, it was positively correlated with Cd, Hg, Pb and Sb [66].

#### 3.2.2. Essential Elements and Oxidative Stress in Women with PCOS

There were thirteen studies assessing an association of essential elements with OS in PCOS [55,56,57,58,59,60,61,62,63,64,65,66,67]. Lower serum Zn levels were detected in three studies (66.3 ± 13.2 vs. 78.1 ± 14.7 μg/dL, 84.4 ± 25.5 vs. 99.4 ± 19.9 μg/dL and 1350 (115–3557.5) vs. 1598.4 (1070.3–2781.9) ppb, respectively) [56,61,66]. Higher Cu levels in PCOS women were detected in a study by Kanafchian et al. (0.206 (0.179–0.248) vs. 0.187 (0.154–0.214) mg/dL, respectively) [65], but on contrary, Kirmizi et al. found Cu levels in the PCOS group were lower when compared to healthy controls (1025 (272.7–1436.5) vs. 1103.9 (573.6–1609.7) ppb, respectively) [66]. The association of Se [55,59] and Ca [60,65] with OS was studied in two studies, the association of Cr was determined in three studies [58,63,66] and the association of Mg was determined in four studies [62,64,65,67].

Supplementation therapy could be beneficial, as altered levels of essential elements, observed in some studies in our systematic review, were associated with markers of OS and chronic inflammation in PCOS populations. In vitro, incubation with selenium was associated with lower levels of neutrophil lipid peroxidation and higher GPx activities and GSH levels [55]. Selenium supplementation decreased serum hs-CRP and plasma MDA levels and increased the pregnancy rate [59]. In a study by Kirmizi et al., Zn was associated with decreased MDA levels and TNF-α levels [66], while Zn supplementation reduced serum hs-CRP, protein-carbonyl and MDA levels [57,62]. There were increased plasma TAC levels and downregulation of the gene expression of interleukin-1 (IL-1) and TNF-α in peripheral blood mononuclear cells observed in the PCOS group after Mg-Zn co-supplementation [62]. Chromium supplementation was associated with a reduction in serum hs-CRP [58] and plasma MDA levels [58,63] and with increased TAC levels [58,63]. Increased plasma TAC levels were also observed following vitamin D-K-Ca co-supplementation in a study by Razavi et al. [60]. There was a positive correlation between Cu and TAS found in a study by Kirmizi et al. [66]. Similarly, Cu levels showed a positive correlation with TAC levels in obese PCOS women in a study by Kanafchian [65]. When considering Mg in association with OS in PCOS women, there were increased plasma NO and TAC levels observed after Mg-vitamin E co-supplementation [64]. In addition, increased TAC levels and decreased TNF-α levels were also associated with Mg-melatonin co-supplementation [67]. There was a negative correlation of Mg levels with TAC in the PCOS group without IR in a study by Kanafchian [65].

Regarding metabolic characteristics in PCOS women, we found eleven studies assessing changes in PCOS regarding essential elements [56,57,58,59,60,61,63,64,65,66,67]. Selenium was associated with decreased alopecia, hirsutism and acne [59]. There was a positive correlation between serum Zn levels, the modified Ferriman–Gallwey score [56] and HOMA-IR [61]. A study by Jamilian et al. revealed decreased alopecia and hirsutism in the Zn group [57]. In addition, Zn appeared to be negatively correlated with FBG [66]. Chromium was associated with decreased hirsutism and acne [58]. Chromium was correlated with decreased levels of FBG, serum insulin, HOMA-IR, triacylglycerols (TG), very-low-density lipoproteins and total cholesterol [63]. Magnesium was associated with reduced hirsutism [64,67] and a decrease in weight, BMI and waist circumference [67]. In a study by Kanafchian et al., Mg levels were negatively correlated with HOMA-IR in the PCOS group with insulin resistance [65]. There was a positive correlation of Cu with BMI and the G/I ratio in the obese PCOS group and a positive correlation of Cu with the G/I ratio and QUICKI in the overweight PCOS group [65]. Regarding hormonal biomarkers suggesting the severity of PCOS, there are several placebo-controlled trials. Compared with the placebo, there were lower levels of 17-hydroxyprogesterone detected following 8 weeks of Zn supplementation (−0.62 ± 0.22 vs. 0.31 ± 0.22 ng/mL after adjustment for baseline levels, age and baseline BMI, respectively) [57]. Selenium supplementation for 8 weeks decreased serum dehydroepiandrosterone (DHEA) levels (−0.36 ± 0.73 vs. −0.02 ± 0.41 μg/mL, respectively) compared with the placebo [59]. Considering 8 weeks of intervention, vitamin D-K-calcium co-supplementation resulted in a significant reduction in serum free testosterone (−2.1 ± 1.6 vs. + 0.1 ± 1.0 pg/mL, respectively) and dehydroepiandrosterone sulfate (DHEAS) levels (−0.8 ± 1.0 vs. −0.1 ± 0.5 μg/mL, respectively) compared with the placebo [60]. There was a higher pregnancy rate observed after Se supplementation [59].

**Figure 3:**
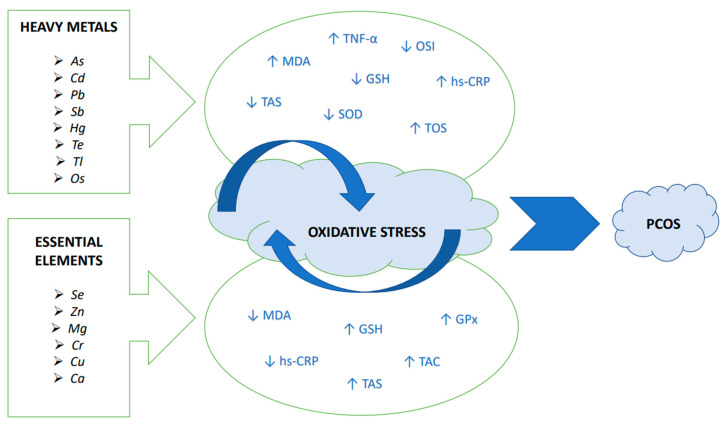
The association between heavy metals and essential elements with OS in PCOS. Abbreviations: As, arsenic; Ca, calcium; Cd, cadmium; Cr, chromium; Cu, copper; GPx, glutathione peroxidase; GSH, reduced glutathione; Hg, mercury; hs-CRP, high sensitivity C-reactive protein; MDA, malondialdehyde; Mg, magnesium; Os, osmium; OSI, oxidative stress index (TOS/TAS ratio); Pb, lead; PCOS, polycystic ovary syndrome; Sb, antimony; Se, selenium; SOD, superoxide dismutase; TAC, total antioxidant capacity; TAS, serum total antioxidant status; Te, tellurium; Tl, thallium; TNF-α, tumour necrosis factor alpha; TOS, total oxidant status; Zn, zinc.

Figure 3 summarizes the association between heavy metals and essential elements with OS in PCOS. 

## 4. Discussion

In general, there is little relevant research on this topic, so it was only possible to include 15 articles. Nevertheless, some interesting connections emerged.

### 4.1. Heavy Metals and Oxidative Stress in Women with PCOS

Significantly higher levels of Cd, Pb and As in PCOS women, found in our literature review [66,68], are in contrast with the study of Zheng et al. in the Chinese population, where no differences in the PCOS population were found regarding Cd, Pb and As serum levels [23]. Similarly, Kurdoglu et al. reported no significant differences in Cd levels and decreased Pb levels among these females [19]. The discrepancy in Cd levels is intriguing, as Cd exposure occurs primarily due to cigarette smoke, and for non-smokers, exposure is mainly from consuming shellfish and food grown in Cd-contaminated soil [70]. In studies by Kirmizi et al. [66] and Zheng et al. [23], smokers were excluded from the study; therefore, one could speculate that other factors such as environmental exposure or individual genetic characteristics can contribute to the difference in Cd levels measured.

Significantly higher inflammatory and oxidative damage in PCOS women, found in our review, is in line observations made by Hilali et al., who reported increased OS in PCOS women [71]. Various markers assessing OS in PCOS, e.g., TAS, TAC, OSI, MDA, SOD and GSH, were evaluated in studies in our systematic review. Diminished GSH levels in PCOS women were found in a study by Dinger et al., although they did not study the possible association with heavy metals [72]. There is still conflicting evidence regarding SOD activities in the PCOS population. Some studies confirmed diminished levels [44,73,74], while Kuşçu et al. found SOD activities to be significantly higher in non-obese PCOS women when compared to age- and BMI-matched controls [75]. Differences between the study results might have been due to different body compensatory responses to higher circulating levels of oxidants, and differences in lifestyle or diet. However, a meta-analysis by Talat et al. revealed statistically significant higher SOD activities in women with PCOS when serum was used as a sample source. According to the authors, this could be due to a smaller number of studies reporting SOD activities in follicular fluid which is, however, considered to be more relevant to assessing the biomarker by providing an essential external microenvironment for oocyte development [76]. Follicular fluid MDA levels were shown to be significantly associated with embryo quality indicators in PCOS-related infertility [18], while follicular fluid TAC levels in PCOS women showed conflicting results [18,77].

Although the role of Tl in PCOS remains unexplored, in humans, higher Tl levels are associated with premature ovarian insufficiency and lower sperm motility, and in zebrafish, with increased atretic follicles and degenerated oocytes and decreased mature oocytes [35]. A study by Padilla et al. revealed a positive correlation of Tl with BMI and waist circumference, common features in PCOS, presumably by inducing OS and thus increasing lipogenesis at the expense of energy production [18,21,77]. In addition, the relationship between plasma Sb, Cd, Pb and Hg and markers of OS, chronic inflammation and impaired glucose homeostasis found in our review suggests possible Sb-, Cd-, Hg- and Pb-induced damage by interfering with oxidative pathways which is in turn related to metabolic problems in PCOS, particularly IR [66,68]. Insulin resistance contributes to hyperandrogenaemia and since Cd and Pb have a high impact on glucose metabolism, it is not surprising that higher Cd, Pb, hs-CRP and TNF-α levels were found in the hirsutism group [66]. This could also partially explain the positive relationship of Cd, Hg, Pb and Sb with the waist/hip ratio or BMI [68]. On contrary, in a study by Padilla et al., Cd and Pb were negatively correlated with BMI and the waist circumference, which might be misleading due to a cross-sectional study design assessing both metals and outcomes at the same time, with further prospective studies needed to address the temporality of these findings [21]. In some studies, a significant relationship was found between urine Sb levels, HOMA-IR and diabetes in both diabetic and non-diabetic patients [78], and an increased risk of gestational diabetes and impaired blood glucose homeostasis in pregnant women [79] was observed. An increased incidence of spontaneous abortions and menstrual cycle disturbances in women working at an antimony metallurgical plant indicates the possible endocrine-disrupting properties of Sb [31]. In some studies, urinary Sb, Cd and Pb levels in non-PCOS populations were associated with FBG, impaired glucose tolerance or type 2 diabetes [80,81]. An increased total Hg exposure may also augment the risk of diabetes and metabolic syndrome, but the lack of consistent epidemiological evidence prevents a conclusion on the causal relationship [30].

### 4.2. Essential Elements and Oxidative Stress in Women with PCOS

Lower Zn levels in PCOS women, found in our review, are in line with some studies [23,43,44], but not with all [19]. Although there were conflicting results regarding Cu levels [65,66], several meta-analyses revealed significantly higher Cu concentrations in PCOS women when compared to healthy controls [24,25,82]. In PCOS, Cu-induced ROS generation in response to hyperglycaemia could serve as an inflammatory trigger for the induction of IR and obesity [12,23]. This was observed in a study by Kanafchian et al., where serum Cu levels were positively correlated with BMI in obese PCOS patients and with IR indices in overweight PCOS women [65]. Similarly, Chakraborty et al. reported significantly higher serum Cu and Zn levels in PCOS women with IR [46] and there was a positive association of Cu levels with BMI, the waist/hip ratio and insulin metabolism parameters [44]. On the other hand, Cu deficiency, presumably due to alterations in lipid metabolism, could increase adiposity [21], and there was an inverse relationship between serum Cu and BMI found in a study by Kurdoglu et al. [19]. However, in the latter study, there was a small sample of participants and PCOS patients were non-obese with no difference between the study and control group regarding BMI.

Altered levels of various markers of OS and metabolic characteristics were observed in our review also with Zn, Cr and Se levels. The negative correlation of Zn with MDA, TNF-α and FBG, found in our review, can be explained by its active role in insulin release, modulation of the inflammatory system and reduction in TNF-α and Il-1 release by inhibiting the proinflammatory response [66]. Significantly lower SOD1, Zn and Cu levels in PCOS women in a study by Bizoń et al. suggest that not only hormonal and metabolic disorders might be directly associated with alterations in SOD1 activity, but also changes in Zn/Cu homeostasis [44]. There are conflicting results regarding the correlation between HOMA-IR and Zn levels [43,61]. Concerning IR and OS, Zn plays a critical role in the function of metalloproteins, participates in insulin metabolism and acts along with Cu in the functions of SOD and CAT [61]. Zinc deficiency could lead to biochemical features of PCOS via decreasing the antioxidant capacity, causing insulin resistance and apoptosis [56]. Moreover, a negative association of serum Zn with BMI and TG, found in a study by Guler et al., suggests a potential benefit of Zn supplement therapy in PCOS women in the prevention of long-term metabolic complications [56]. Hyperhomocysteinaemia can further contribute to increasing the cardiovascular risk in PCOS women [83]. Lower homocysteine levels in PCOS women found by Guler et al. can be explained with the small number of subjects included in their study [56]. In our review, Zn supplementation appeared to be effective in reducing OS, alopecia and hirsutism [57]. This is in line with the reduced OS in diabetic populations [84] and with reports of improved hair growth in humans [85], which might be the result of the ability of Zn to inhibit the hair follicle regression and precipitate hair follicle recovery, as observed in mice [86]. In addition, our review also revealed decreased OS and chronic inflammation markers, hirsutism and acne in PCOS women with Cr supplementation [58,63]. This is supported by some animal models, where Cr intake resulted in a significant decrease in CRP concentrations in diabetic rats [87] and MDA concentrations in rats fed a high-fat diet [88], and in an increase in serum TAC in growing pigs [89]. The exact mechanism by which Cr intake may decrease CRP is not known, but it could be partly explained by interfering with OS pathways [49]. Decreased markers of OS following Cr intake might be linked to the decreased action of epinephrine due to the insulinotropic effect of Cr and activation of GSH reductase or other enzymes detoxifying free radicals and ROS [49,90]. In our review, Cr supplementation in PCOS women also appeared to improve glycaemic control and markers of cardio-metabolic risk [63]. This is partially in line with a study by Jamilian in 2015, where the Cr intake had a beneficial impact on markers of insulin metabolism [91]. In PCOS mice, Cr supplementation significantly improved FBG and insulin levels [92]. Chakraborty et al. reported significantly lower Cr levels in PCOS women with IR with a correlation between Cr levels and fasting insulin levels in PCOS-associated IR [46]. Taking Cr supplements improved TG and HDL levels [93] as well as total cholesterol levels [94] in patients with type 2 diabetes mellitus. It has been speculated that Cr intake may improve lipid profiles through reducing TG synthesis (as seen in sheep) and elevating insulin sensitivity, as insulin is a central regulator of lipid homeostasis through enhancing TG synthesis [63,95].

In our review, Se supplementation was associated with decreased hirsutism, acne and alopecia, and significant reductions in OS and serum DHEA [55,59]. Se intake in women with PCOS had also beneficial effects on insulin metabolism parameters [40]. Moreover, a study by Coskun et al. demonstrated a negative correlation between Se, LH and total testosterone, suggesting that decreased Se levels in the PCOS population may be related to hyperandrogenism [96]. On the contrary, Mohammad Hosseinzadeh et al. reported that Se supplementation may worsen insulin resistance in PCOS patients, possibly due to an elevation in blood selenoprotein P levels, which in turn might have an adverse effect on insulin metabolism and IR. The authors concluded that indiscriminate consumption of Se supplements warrants caution until the results of larger studies become available [97]. Regarding the favourable effects of Se intake on female fertility, possibly due to hormonal changes, an improved ovulatory function and improved markers of insulin metabolism and OS were observed, which in turn may result in improved fertility. There was a positive association with higher pregnancy rates in PCOS women [59], improved conception rates in ewes [98], improved fertility in cattle [99] and lower levels of serum and follicular fluid Se in women undergoing in vitro fertilization treatment compared to nonpregnant control women [100].

We found two studies assessing Ca levels in PCOS populations in our systematic review [60,65], with a significant reduction in OS, serum free testosterone and DHEAS demonstrated in vitamin-D-deficient PCOS women following vitamin D-K-Ca co-supplementation [60]. This is in accordance with decreased total testosterone and androstenedione levels in a study by Pal et al. after vitamin D and Ca co-supplementation among overweight PCOS patients. Since IR parameters were unaltered in their study population, the direct effects of vitamin D and Ca supplementation on the steroidogenesis pathway (ovarian and/or adrenal) can be hypothesized to explain the observed reduction in circulating androgens [101]. Similarly, vitamin D-Ca co-administration in PCOS women resulted in improved follicular maturation, regularity of menses and androgen-related symptoms, particularly in vitamin-D-deficient women [102]. Reduced OS following vitamin D and Ca co-supplementation was observed also among patients with gestational diabetes in a study by Asemi et al. [103], hypothesizing that joint vitamin D, K and Ca supplementation might reduce the generation of OS more than either vitamin D, vitamin K or Ca alone [60]. Regarding Mg supplementation, there was an association with reduced OS and markers of chronic inflammation (e.g., hs-CRP) observed in four studies of our systematic review [62,64,67]. Although Kanafchian et al. found a negative correlation between Mg and TAC levels in non-IR PCOS patients, the authors pointed out small size samples, decreasing the reliability of the results [65]. Nevertheless, decreased CRP levels after Mg supplementation are in line with a meta-analysis conducted by Simental-Mendia in people with high levels of inflammation [104]. The beneficial effect of Mg supplementation was seen also in suppressed cytokine/chemokine levels in amniotic fluid and placentas in rats [105]. Magnesium intake may decrease inflammatory factors due to its antagonism to Ca, which plays an important role in inflammation. In addition, insulin secretion is a calcium-dependent process and Ca levels are in turn interrelated to those of Mg. Magnesium deficiency can lead to changes in cellular Ca levels which appeared to be associated with IR and diabetes, common features in PCOS [46]. PCOS women with IR exhibited significantly lower Mg and significantly higher Ca serum levels; in PCOS-associated insulin resistance, serum Mg levels were significantly correlated with fasting insulin levels [46]. Mg supplementation was also associated with reduced hirsutism in PCOS women [64,67], pointing out the possible beneficial antioxidant and anti-inflammatory characteristics of Mg and improved insulin sensitivity [106]. On the other hand, Sharifi et al. concluded that serum Ca concentrations when compared to Mg levels tend to be a more potent predictor of PCOS and related to IR [107]. A decrease in weight, BMI and waist circumference observed after Mg-melatonin co-supplementation indicates the ameliorating effect of the antioxidative properties of melatonin and Mg on the pro-inflammatory state seen in PCOS, and therefore they have beneficial effects on obesity and adipokine patterns leading to IR [67].

## 5. Conclusions

Altered levels of heavy metals and essential elements have been associated with the development of OS and metabolic and hormonal changes in PCOS women. In this systematic review, we summarized the knowledge on the association of heavy metals and essential elements with OS in PCOS in the last 15 years. Studies revealed that PCOS women had increased Sb, Cd, Pb, Hg, As, Te, Tl and Os blood levels and decreased Zn blood levels, while the results of Cu blood levels were conflicting. Some studies showed a significant correlation between heavy metals (Sb, Cd, Pb, Hg, As, Te and Tl) and essential elements (Se, Zn, Cr, Ca, Mg and Cu) and markers of OS and chronic inflammation. When considering metabolic and hormonal characteristics in PCOS, there were significant associations regarding heavy metals (Sb, Cd, Pb and Hg) as well as essential elements (Zn, Cr, Se, Ca, Mg and Cu). Several studies in our review indicated the possible benefits from supplementation therapy (Zn, Cr, Se, Ca, vitamin D-K-Ca and Mg-melatonin co-supplementation) in reducing OS and endocrinological problems related to PCOS, such as hirsutism, acne, hyperandrogenaemia, obesity and IR. Our findings therefore confirmed an association between heavy metals and essential elements with OS in PCOS women, which in turn might be related to various endocrinological characteristics commonly seen in PCOS.

However, we discovered that there was a plethora of different markers used to define OS in our studies. Some study designs did not include healthy controls because the supplementation impact in a PCOS group was observed. There was also a small number of women included in each study. This made it challenging to compare the results and provide proper conclusions. In the future, larger studies involving a large sample size, possibly with long-term monitoring, are needed for clear elucidation of the role of heavy metals and essential elements in PCOS in relation to OS. There has been a rapidly growing interest in OS, antioxidant supplementation and a healthy lifestyle observed in recent years. According to our opinion, it is therefore of high importance to provide as precise as possible conclusions of clinical studies to the public to avoid misunderstanding, and even more importantly to avoid data manipulation. Further research on a larger number of patients is needed to be able to answer these important questions.

## Figures and Tables

**Table 1:** Association of heavy metals and essential elements with oxidative stress in polycystic ovary syndrome (PCOS). Studies included in this systematic review. Statistically significant differences are presented.

Study, Year, (Country)	Study Design	Participants (No. of Women)	Sample Type	Exposure	Main Conclusions
Köse et al. (2014), Turkey [55]	Case–control study	20: 10 with PCOS (mean age: 24.0 ± 5.5), 10 controls (mean age: 27.8 ± 6.9)	Peripheral whole blood sample	Se	- Lower LP levels in PCOS + CPZ, PCOS + Se and PCOS + Se + CPZ groups - Higher GPx activity and GSH level in PCOS + Se and PCOS + Se + CPZ groups - Lower increase in Ca^2+^ in PCOS + Se and PCOS + Se + CPZ groups - Lower increase in Ca^2+^ when exposed to CPZ and Se - Greater effect of CPZ + Se on Ca^2+^ than CPZ
Guler et al. (2014), Turkey [56]	Prospective study	86: 53 with PCOS (mean age: 25.4 ± 6.7), 33 controls (mean age: 28 ± 5.9)	Overnight fasting venous blood sample	Zn	- Lower mean Zn and homocysteine levels in PCOS - Negative correlation of serum Zn with BMI and TG - Serum Zn is the only significant and independent metabolic variable predicting PCOS - Lower mean Zn levels in lean PCOS group compared to lean control group - Positive correlation between serum Zn level and modified Ferriman–Gallwey score
Jamilian et al. (2015), Iran [57]	Randomized, double-blind, placebo-controlled study	48 with PCOS aged 18–40	Fasting blood sample	Zn	- Decreased alopecia and hirsutism in Zn group - Decreased MDA levels in Zn group - Higher TAC levels and lower 17-OH-P in Zn group after adjustment for baseline levels, BMI and age
Jamilian et al. (2015), Iran [58]	Randomized, double-blind, placebo-controlled study	60 with PCOS aged 18–40	Fasting blood sample	Cr	- Decreased hirsutism and acne in Cr group - Decreased serum hs-CRP and plasma MDA levels in Cr group - Higher TAC levels in Cr group - Lower PRL and NO levels and higher LH levels after adjustment for baseline levels of biochemical variables, BMI and age
Razavi et al. (2016), Iran [59]	Randomized, double-blind, placebo-controlled study	64 with PCOS aged 18–40	Fasting blood sample	Se	- Higher pregnancy rate in Se group - Decreased alopecia, hirsutism and acne in Se group - Decreased serum DHEA, serum hs-CRP and plasma MDA levels in Se group - Lower FSH levels after adjustment for age and baseline BMI
Razavi et al. (2016), Iran [60]	Randomized, double-blind, placebo-controlled study	60 vitamin D deficient with PCOS aged 18–40	Fasting blood sample	Ca	- Decreased serum free T, DHEAS and plasma MDA levels in vitamin D-K-Ca group - Increased plasma TAC levels in vitamin D-K-Ca group - Decreased LH and hs-CRP levels after adjustment for baseline values of biochemical parameters, age and baseline BMI
Özer et al. (2016), Turkey [61]	Descriptive study	124 aged 20–30: 71 with PCOS, 53 controls	Venous blood sample	Zn, Cu	- Lower serum levels of Zn and CAT activity in PCOS group - Higher MDA and GPx levels in PCOS group - Lower serum levels of Zn, CAT activity and MDA in GPx levels in PCOS group with IR (when compared to healthy controls) - Lower serum Zn levels and CAT activity and higher MDA levels in PCOS group with IR (when compared to PCOS group without IR) - Lower serum Zn levels and CAT activity and higher MDA and GPx levels in infertile PCOS group (when compared to healthy controls) - Lower serum Zn levels and CAT activity and higher MDA levels in infertile PCOS group (when compared to fertile PCOS group) - Positive correlation between HOMA-IR and Zn levels
Ebrahimi et al. (2017), Iran [62]	Randomized double-blind, placebo-controlled tri-al	60 with PCOS aged 18–40	Fasting blood sample	Mg, Zn	- Decreased serum hs-CRP and protein carbonyl in Mg-Zn group - Increased plasma TAC levels in Mg-Zn group - Downregulation of gene expression of IL-1 and TNF-α in peripheral blood mononuclear cells in Mg-Zn group
Jamilian et al. (2018), Iran [63]	Randomized, double-blind, placebo-controlled study	40 infertile with PCOS, candidate for IVF, aged 18–40	Fasting blood sample	Cr	- Decreased FBG, serum insulin, HOMA-IR, TG, VLDL and total cholesterol levels and increased QUICKI in Cr group - Increased plasma TAC levels and decreased MDA levels in Cr group
Shokrpour et al. (2019), Iran [64]	Randomized, double-blind, placebo-controlled study	60 with PCOS aged 18–40	Fasting blood sample	Mg	- Reduced hirsutism and serum hs-CRP in Mg-vitamin E group - Increased plasma NO and TAC in Mg-vitamin E group
Kanafchian et al. (2019), Iran [65]	Case–control study	150 aged 20–40: 60 with PCOS, 90 controls	Fasting venous blood	Cu, Mg, Ca	- Higher serum Cu levels in PCOS group - Negative correlation of serum Mg levels with HOMA-IR in PCOS group with IR - Positive correlation of serum Mg levels with QUICKI in PCOS group with IR - Negative correlation of serum Mg levels with TAC levels in PCOS group without IR - Positive correlation of serum Cu levels with BMI, TAC and G/I ratio in obese PCOS group - Positive correlation of serum Cu levels with G/I ratio and QUICKI in overweight PCOS group
Kirmizi et al. (2020), Turkey [66]	Case–control study	154: 84 with PCOS (aged 22–36), 70 controls (aged 21–39)	Blood sample	As, Cr, Cd, Sb, Hg, Pb, Cu, Zn	- Higher plasma Sb, Cd, Pb and Hg levels in PCOS group - Lower Zn and Cu levels in PCOS group - Lower TAS, OSI and SOD values in PCOS group - Higher TOS, MDA, hs-CRP and TNF-α levels in PCOS group - Positive correlation between Cu and TAS in PCOS group - Negative correlation between Zn and MDA, TNF-α and FBG in PCOS group - Positive correlation between Sb and MDA, TNF-α, HOMA-IR and FBG; negative correlation with TAS, OSI and SOD in PCOS group - Positive correlation between Cd and MDA, hs-CRP, BMI, HOMA-IR, FBG and insulin levels; negative correlation with TAS in PCOS group - Positive correlation between Pb and FBG, MDA, TOS and hs-CRP; negative correlation with TAS, OSI and SOD in PCOS group - Positive correlation between Cd, Hg, Pb and Sb and waist/hip ratio in PCOS group - Higher Cd, Pb, TNF-α and hs-CRP levels in hirsutism group
Mousavi et al. (2021), Iran [67]	Randomized, double-blind, placebo-controlled study	84 with PCOS aged 18–40	Blood sample	Mg	- Decrease in weight, BMI, waist circumference, hirsutism and serum TNF-α levels in Mg-melatonin group - Decrease in waist circumference in Mg group - Increase in TAC levels in Mg-melatonin group
Abudawood (2021), Saudi Arabia [68]	Prospective study	106 aged 19–35: 56 with PCOS, 50 controls	Blood serum	As, Cd, Pb, Hg	- Increased serum levels of As, Cd, Pb and Hg in PCOS group - Negative correlation of As and Pb with SOD and GSH levels in PCOS group - Negative correlation of Hg with GSH levels in PCOS women - Positive correlation of As with increased Cd, Pb and Hg levels in PCOS group - Significant correlations between Cd an Pb and between Cd and Hg - Positive correlation of Hg with FBG and HbA1c in PCOS group - Positive correlation of Cd with total cholesterol in PCOS group
Abudawood (2023), Saudi Arabia [69]	Case–control study	106: 50 with PCOS (mean age: 30.41 ± 6.8), 56 controls (mean age: 29.16 ± 6.2)	Blood serum	Te, Tl, Sb, Os	- Increased levels of Te, Tl, Os and Sb in PCOS group - Decreased serum TAC levels in PCOS group - Positive correlation between Te levels and Tl, Os and Sb, and negative correlation with TAC levels in PCOS group - Positive correlation between Tl and Sb, Sb and Os, and negative correlation between Tl, Sb, Os and TAC

Abbreviations: As, arsenic; BMI, body mass index; Ca, calcium; Ca^2+^, neutrophil intracellular calcium concentration; CAT, catalase; Cd, cadmium; CPZ, capsazepine; Cr, chromium; Cu, copper; DHEA, dehydroepiandrosterone; DHEAS, dehydroepiandrosterone sulphate; FBG, fasting blood glucose; FSH, follicle-stimulating hormone; G/I ratio, glucose/insulin ratio; GPx, glutathione peroxidase; GSH, reduced glutathione; HbA1c, glycated haemoglobin; Hg, mercury; HOMA-IR, Homeostatic Model Assessment for Insulin Resistance; hs-CRP, high sensitivity C-reactive protein; IL-1, interleukin-1; IR, insulin resistance; LH, luteinizing hormone; LP, neutrophil lipid peroxidation; MDA, malondialdehyde; Mg, magnesium; NO, nitric oxide; Os, osmium; OSI, oxidative stress index (TOS/TAS ratio); 17-OH-P, 17-hydroxyprogesterone; Pb, lead; PCOS, polycystic ovary syndrome; PRL, prolactin; Sb, antimony; Se, selenium; SHBG, sex hormone-binding globulin; SOD, superoxide dismutase; QUICKI, quantitative insulin sensitivity check index; T, testosterone; TAC, total antioxidant capacity; TAS, serum total antioxidant status; Te, tellurium; TG, triacylglycerols; Tl, thallium; TNF-α, tumour necrosis factor alpha; TOS, total oxidant status; VLDL, very-low-density lipoprotein; Zn, zinc.

## Data Availability

The data are contained within the article.

## Supplementary Materials

The following supporting information can be downloaded at: https://www.mdpi.com/article/10.3390/antiox12071398/s1, PRISMA 2020 Checklist. Ref. [108] is cited in the Supplementary Materials.
